# Helium Isotopes Quantum Sieving through Graphtriyne Membranes

**DOI:** 10.3390/nano11010073

**Published:** 2020-12-31

**Authors:** Marta I. Hernández, Massimiliano Bartolomei, José Campos-Martínez

**Affiliations:** Instituto de Física Fundamental, Consejo Superior de Investigaciones Científicas (IFF-CSIC) Serrano 123, 28006 Madrid, Spain; marta@iff.csic.es (M.I.H.); maxbart@iff.csic.es (M.B.)

**Keywords:** quantum sieving, graphynes, 2D material filters, wave packet calculations, isotope separation

## Abstract

We report accurate quantum calculations of the sieving of Helium atoms by two-dimensional (2D) graphtriyne layers with a new interaction potential. Thermal rate constants and permeances in an ample temperature range are computed and compared for both Helium isotopes. With a pore larger than graphdiyne, the most common member of the γ-graphyne family, it could be expected that the appearance of quantum effects were more limited. We find, however, a strong quantum behavior that can be attributed to the presence of selective adsorption resonances, with a pronounced effect in the low temperature regime. This effect leads to the appearance of some selectivity at very low temperatures and the possibility for the heavier isotope to cross the membrane more efficiently than the lighter, contrarily to what happened with graphdiyne membranes, where the sieving at low energy is predominantly ruled by quantum tunneling. The use of more approximate methods could be not advisable in these situations and prototypical transition state theory treatments might lead to large errors.

## 1. Introduction

Two dimensional (2D) materials have become ubiquitous and are being increasingly used in many technological applications [1,2] with progress in both theory [3] and experiment [4]. The fabrication of different 2D membranes by the conjunction of several so-called top-down approaches as well as bottom-up methods are able to produce a layer with almost any feature at will [2]. From the, nowadays, very famous graphene, a large set of many different compounds are now produced in the form of monoatomic layer 2D materials. Among all of these, graphynes and within them, γ-graphynes [5,6] are very popular since several routes to fabrication have been developed and many possible technological applications have been envisaged [7]. These materials are made solely of carbon atoms bonded by sp and $sp^{2}$ hybridizations. In simple words they consist of benzene rings joined by *n*-acetylenic groups, giving rise to increasing nanopore sizes in graphdiyne for n=2, graphtriyne for n=3 and so on. Thus, as an example of the fruitful interplay between theory and experiment previously mentioned, in this family graphdiyne was first proposed theoretically [8] and synthesized later on [9] for the first time, some years before new procedures were designed [10,11] and the route to lab fabrication became quite regular and common [4], with the theoretical work always side-by-side as an invaluable tool [12,13].

Among the multiple applications [7], 2D materials were early proposed as efficient sieves at the molecular level [14,15,16], and in this context the graphyne family was one of the suitable candidates due to the regularity in the position and size of the nanopores. Particularly, it has been suggested that these membranes could be efficiently used for the isotopic separation of several atomic or molecular species [17,18,19], a process which continues being nowadays very challenging and troublesome. Helium is a scarce resource, mostly obtained from natural gas, from where it needs to be separated from the other components. It is mostly used for applications related to technology and the lightest isotope, ${}^{3}$He, is even scarcer, yet it is critical for several technologies, and this demand produces from time to time a shortage in the supplies of this species [20,21]. The need for isotopic separation in other simple gases is also important, as for example in the case of hydrogen [22], so the need for efficient and cheap filters at the molecular level is expected to increase in the near feature.

For low enough pressures, the study of the separation of different compounds from a gas mixture can be modeled as the dynamics of an atom or molecule interacting with the 2D membrane. At low temperatures, it is expected that quantum effects may play an important role leading to larger selectivity ratios. Recently, a rather large number of works have shown that quantum tunneling might rule an efficient separation process [18,23,24,25,26,27,28,29]. While the quantum nature of the processes was stressed, most of the calculations relied on classical mechanics and/or combinations of one-dimensional calculations as well as some estimations using Transition State Theory (TST) [26,30,31] based on the calculation of the zero point energy (ZPE) along the 2D nanopore directions, that has also been shown to be of importance by theory [15,32,33,34,35] and experiment [36,37].

It is important to emphasize that tunneling and ZPE quantum phenomena affect different isotopes in opposite directions, thus on the one hand, the lighter atom or molecule will overcome more efficiently a barrier by tunneling than the heavier one. On the other hand, the latter will have a lower ZPE along the membrane nanopore and therefore will cross more efficiently to the other side of the layer. We have shown in graphdiyne [26] the relation and relative importance of these two effects. More recently [38], the limits of more approximated treatments, such as one-dimensional tunneling and TST, were assessed by means of three-dimensional wave packet calculations. We found that one possible scenario for these popular methodologies to breakdown was that in which there were no barrier to impede atoms crossing the membrane, a case that, as we will see below, is just happening in graphtriyne for He atoms.

We here present quantum-mechanical calculations for the transmission of He atoms through a periodic and rigid one-atom-thick graphtriyne membrane, with special emphasis in the low energy regime, and the effect that could be shown in the behavior of its isotopes (${}^{3}He,{\;}^{4}He$). Graphtriyne is the next member of γ-graphyne family to the very much studied graphdiyne, and to the best of our knowledge it has not been synthesized yet, although the next one by the number of acetylenic groups, graphtetrayne, has been very recently reported as successfully synthesized [39,40]. Although it appears that odd members of the graphyne family could be more difficult to prepare in the lab, one can not completely rule out the possibility of a novel procedure that could reach a successful synthesis of this material. Therefore, our aim in the study of graphtriyne is twofold. On the one hand, we want to predict what one can expect of the behavior and properties of such a material, which presents a nanopore certainly larger than graphdiyne and, on the other hand, to investigate how the properties change as the nanopore increases its size. We anticipate that the behavior is far from simple and that a strong quantum behavior is present at low energies; which should be taken into account in future studies whether theoretical or experimental. As in previous works [38] our methodology combines wavepacket propagation by a periodic surface [41] and the calculation of transmission probabilities using a flux surface [42,43] with a new and accurate potential energy surface computed with a procedure that has already proven to be reliable [44]. After that, thermal rate coefficients and the more common, in this context, permeance magnitudes will be computed and discussed.

The paper is organized as follows. In the next Section we present the system with the interaction potential and dynamical method to study the transmission of a 3D wave packet through a periodic membrane. Results are presented and discussed in Section 3, and finally in Section 4 we end with conclusion and perspectives.

## 2. Theoretical Approach

**Figure 1:**
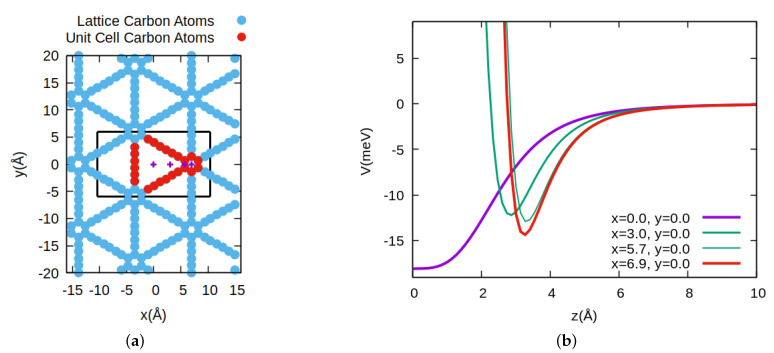
(**a**) Graphtriyne lattice. The red filled points represent the atoms of the unit cell and the rectangle indicates the (x,y) grid for the wave packet propagation. Crossed points indicate *z* directions at which the interaction potential is computed, as shown in in the right panel. (**b**) Interaction potential along the *z* coordinate at several (x,y) positions indicated in the lower right part, and by crossed points in the left panel.

We consider the collision of a He atom with a non-vibrating periodic single monolayer, that is, graphtriyne, and we describe the process by solving the Time-Dependent Schödinger Equation (TDSE), once a suitable interaction potential has been computed. The lattice is represented in Figure 1a, where the atoms belonging to the non primitive (rhomboid) unit cell are represented by red filled points. The unit cell is slightly different to the one commonly used [45] in that it includes the middle of the pore. The reason for this choice is to facilitate the use of a rectangular periodic unit, plotted as a black rectangle in the same figure, to ease the quantum calculation on a rectangular grid, as explained below. When the He is in a given (x,y,z) position it feels the 2D layer and in Figure 1b we show how is this interaction as we approach the graphtriyne membrane in the direction (*z*) perpendicular to the surface, in four different positions of the unit cell. Since the nanopore is large enough for the atom size, there is no barrier to surmount when we are approaching by the middle of the pore. This interaction potential, calculated as indicated below, decreases the well and then an expected barrier appears as we move out of the center of the pore.

### 2.1. The Interaction Potential

The interaction potential is given by a sum of pairwise interactions between the He and the carbon atoms present in the layer. These atom-atom interactions, depending on *r*, the distance between the rare gas atom and those in the lattice, are expressed in the so-called Improved Lennard-Jones (ILJ) [46] formula given by

(1) $$V\left(r\right)=\varepsilon \left(\frac{6}{n(r)-6}{\left(\frac{R_{m}}{r}\right)}^{n(r)}-\frac{n(r)}{n(r)-6}{\left(\frac{R_{m}}{r}\right)}^{6}\right),$$

where, ϵ and $R_{m}$ are the usual well depth and equilibrium distance and

(2) $$n\left(r\right)=\beta +4{\left(\frac{r}{R_{m}}\right)}^{2}.$$

The parameters of this pair potential were optimized from comparison with benchmark estimations of interactions energies, obtained at the “coupled” supermolecular second-order Møller-Plesset perturbation level of theory [47], by using aug-cc-pVTZ and aug-cc-pV5Z basis sets for the carbon and helium atoms, respectively, and following the guidelines described in detail in Reference [25]. The values of the parameters are thus given in Table 1.

### 2.2. Wave Packet Propagation

The (three-dimensional) wave packet is propagated following the prescriptions given in our early work [38], by means of the Split-Operator method [48,49]. The membrane spreads along xy coordinates, and the atom of mass μ is initially represented by a Gaussian wave-packet [50] in the *z* direction and a plane wave in the remaining (x,y) degrees of freedom. In these conditions the position of the atom is given by r=(R,z), *z* being the distance to the membrane plane and R=(x,y). The wave packet is discretized on a grid of evenly spaced (x,y,z) points. To take advantage of the lattice periodicity, the plane wave in the direction parallel to the surface is prepared with a wave vector K in R, matching the size of the (x,y) grid to the unit cell [41], $\left(\Delta_{x},\Delta_{y}\right)$. Finally, since the propagation at low energies needs to be carried out for a long period of time, the wave packet is absorbed at the *z*-edge boundaries [51,52] by a damping function, to avoid artificial reflections. Computational details of the initial wave packet and propagation conditions are given in the Appendix A.

The initial wave packet which represents an incident plane wave with a kinetic energy $E=\frac{\hbar^{2}k^{2}}{2\mu }$ and a corresponding wave vector $k=(k_{z},K)$ is split in a transmitted and a reflected wave after reaching the membrane. The scattering of the wave packet is elastic since there is no exchange of energy with the membrane, and the parallel wave vectors of these waves obey the Bragg condition whereas the perpendicular one is modified to satisfy conservation of energy,

(3) $$k_{z,G}^{\pm }=\pm {\left[k^{2}-{\left(K+G\right)}^{2}\right]}^{1/2},$$

where G is a reciprocal lattice vector (see Reference [38] for details). In these conditions, the probability of transmission, P(E), through a surface $z=z_{f}$ separating transmitted from incident and reflected waves [42], is given by

(4) P(E)=2πℏ2μIm∫dxdyΨE+*(x,y,zf)dΨE+dz∣z=zf,

where $\Psi_{E}^{+*}\left(x,y,z_{f}\right)$ is obtained from the time-energy Fourier transform of the evolving wave packet [43,53].

The transmission rate coefficient is then obtained from the integration of P(E), properly weighted by the Boltzmann factor:

(5) $$R\left(T\right)=\frac{1}{hQ_{trans}}\int e^{-E/(k_{B}T)}P\left(E\right)dE,$$

where we have changed the more traditional K(T) by R(T) to avoid confusions with the wave vectors “*k*” and where $Q_{trans}={\left(2\pi \mu k_{B}T/h^{2}\right)}^{3/2}$ is the translational partition function per unit volume, with $k_{B}$ and *h* the Boltzmann and Planck constants respectively. In detail, P(E) would not only depend on the translational energy but also of the incident angle, that is, the parallel wave vector K. We are reporting here the results corresponding to a perpendicular approach (**K** = **0**). The effect of varying the angle of incidence (**K** ≠ **0**) would be that of decreasing the value of P(E) so in a sense the values reported here should be considered an upper limit.

For the sake of completeness, we have also computed the permeances [54] (effective fluxes per pressure unit in GPU units ($1\;GPU=3.35\times 10^{-10}$ mol/(m${}^{2}$·s·Pa)), a magnitude that is more commonly used in sieving and filtering research. This magnitude is computed as [15,23],

(6) $$S\left(T\right)=\frac{{\langle P\rangle }_{T}}{{\left(2\pi \mu k_{B}T\right)}^{1/2}},$$

where ${\langle P\rangle }_{T}$ is the thermal average of the probability

(7) $${\langle P\rangle }_{T}={\left(\frac{\mu }{2\pi k_{B}T}\right)}^{1/2}\int_{0}^{\infty }P\left(v_{z}\right)\;\exp\left(\frac{-\mu v_{z}^{2}}{2k_{B}T}\right)\;dv_{z}$$

and $v_{z}={\left(2E/\mu \right)}^{1/2}$ is the velocity of the atom impinging perpendicularly upon the surface.

Finally, for both thermal rate coefficients and permeances, selectivity is defined as the ratio between the values corresponding to each isotope, that is, for isotopes *A* and *B*, and corresponding rates (or permeances) $M_{A}$ and $M_{B}$, the selectivity $S_{A/B}$ is,

(8) $$S_{A/B}\left(T\right)=\frac{M_{A}\left(T\right)}{M_{B}\left(T\right)}.$$

## 3. Results and Discussion

**Figure 2:**
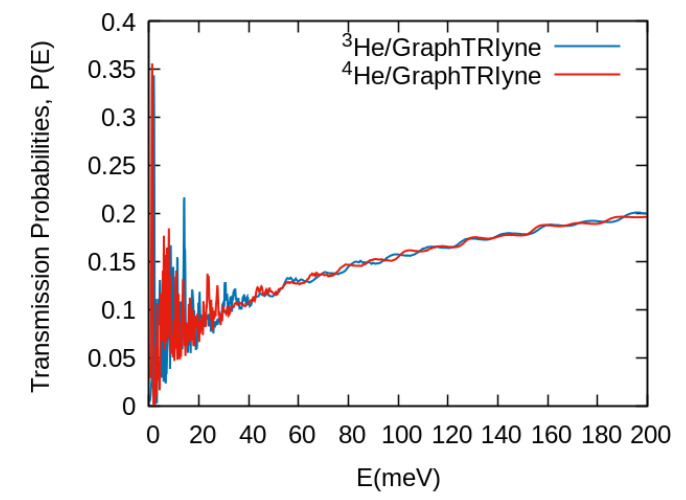
Transmission probabilities of ${}^{3}He$ (blue) and ${}^{4}He$ (red) as a function of the kinetic energy of the atoms through graphtriyne.

**Figure 3:**
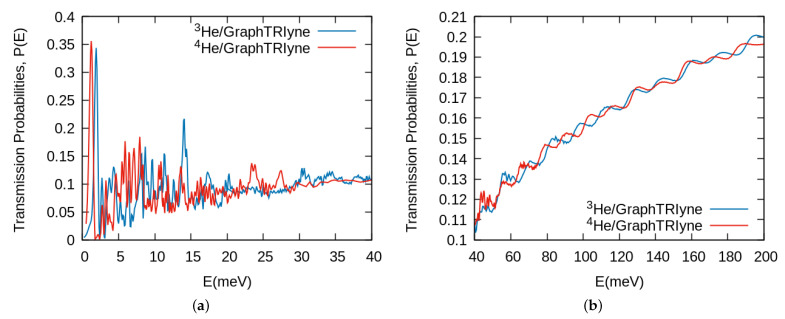
${}^{4}$He and ${}^{3}$He transmission probabilities as a function of the kinetic energy in (**a**) low energy regime, (**b**) at higher kinetic energies.

Once the interaction potential has been presented and following the procedure described above we have computed the transmission probabilities over a wide range of initial kinetic energies. The values of the probabilities are shown in Figure 2. Two different regions can be clearly distinguished, one at low energy up to ≈40 meV, and thereafter till the maximum value of incident energy computed. We have to recall that for this material the nanopore is large enough that there is no barrier to impede the passage of He atoms through the middle of it, and that there is a smooth transition till the acetylenic bonds or benzene ring are reached, where a barrier begins to take shape and later becomes impenetrable, as can be seen in Figure 1b. Because of that feature one could expect a smooth behavior already at low energies, with a transmission probability determined by the effective pore size at that energy. However, by no means this is the case. We are presenting in Figure 3a a closer look at the low energy region, where we observe that there is a highly oscillatory behavior with very strong peaks at lower energies, in fact larger than values at the maximum kinetic energies shown in Figure 2. This behavior has been checked by changing many computational features of the wave packet propagation (see Appendix A) to assure that they are not an artifact of the calculations.

We believe the reason for this oscillatory behavior lies upon another quantum phenomenon that is not the mentioned tunneling (remember there is no barrier in the middle of the pore) or the quasi-bound states in the pore along the (x,y) direction, and that this effect can be appreciated at higher energies as we will see later on. We believe that these numerous peaks in the transmission probability in the low energy region (Figure 3a) are due to the so-called selective adsorption resonances, observed in 1930s gas-surface diffraction experiments by Stern and coworkers [55] and first explained by Lennard-Jones and Devonshire [56] as a temporal trapping of the atoms in the adsorption well of the gas-surface potential.

These resonances involve a particular case of the Bragg condition of Equation (3), that is, when a reciprocal lattice vector $G^{\prime }$ produces a transition to a bound state of the laterally averaged interaction potential [57]. This process can be pictured as a transfer of momentum from the perpendicular to the parallel direction resulting in a quasibound state in the perpendicular direction. Due to the corrugation of the interaction potential, this trapped state is eventually diffracted at another lattice vector and the particle is scattered back to the asymptotic region. This feature affects diffraction intensities spectra by producing Lorentzian or Fano-type shapes around the energy where the resonance condition is fulfilled. In the present case of a porous layer, in addition to being scattered back to the z>0 region, the quasibound states can be leaked through the pores and rather scattered forward towards the z<0 asymptotic region, contributing in this way to an enhancement of the transmission probability, as observed in Figure 3a. These resonances are different for both isotopes (${}^{4}He{,}^{3}He$), and are located at different energies. We note that the first very strong peak corresponds to the heavier ${}^{4}He$ atom and that this will affect macroscopic magnitudes as we will next see.

In the higher energy range (Figure 3b) resonant peaks are less pronounced and the behavior of the probabilities becomes somewhat different: they depict a step-like shape, that is, at certain energies probabilities rise rapidly and later they smoothly decrease until the next “step”. These jumps in the transmission probabilities can be understood by the successive population of excited states associated to the bound motions of the transition state, as is discussed in detail in References [26,38].

The results just presented for transmission probabilities suggest that because of the strong influence of the resonances, we can expect some selectivity in the transmission of one isotope against the other. These probabilities are very difficult to converge since it requires very long propagation times. In fact in our case, there is still a small portion of the wave packet in the interaction region that could affect results at extremely low energy, in the region below ≈1 meV.

When comparing grahtriyne with other 2D materials with smaller pores (i.e., graphdiyne), one would expect a larger flux, with a smooth increasing behavior as the temperature rises. However, the presence of a strong resonant behavior at low energies is very interesting since it would allow some selectivity in the quantum sieving, at least at low temperature. We will see how this manifests in more macroscopic magnitudes.

**Figure 4:**
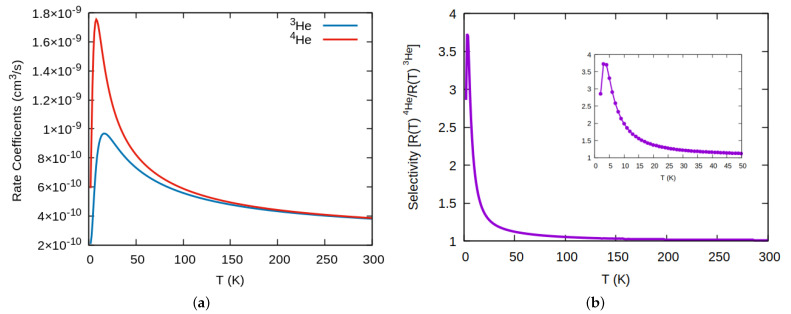
(**a**) Thermal rate coefficients for the Helium graphtriyne system. (**b**) Rate coefficients selectivity ${}^{4}He{/}^{3}He$.

Rate coefficients as functions of temperature are determined from these probabilities (Equation (5)), and are shown for both ${}^{3}He$ and ${}^{4}He$ in Figure 4a. Their values are large as expected due to the large pore area. They present a maximum at low temperature as opposed to more typical behavior where the rate coefficients increase with temperature. More noticeable is the fact that it is the heavier isotope, ${}^{4}He$, the one that presents larger values at low temperature, a different behavior to what was found for graphdiyne [26,38] or related membranes whose behavior at low energy is dominated by the tunneling, that is, the lighter isotope, ${}^{3}He$, is the one that it is favored at low temperature. In Figure 4b, we show the ratio of thermal rates. As a consequence of the previous effects, we find that this system does present a significant selectivity (≈4), although for low temperatures as can be better appreciated in the inset of the figure.

**Figure 5:**
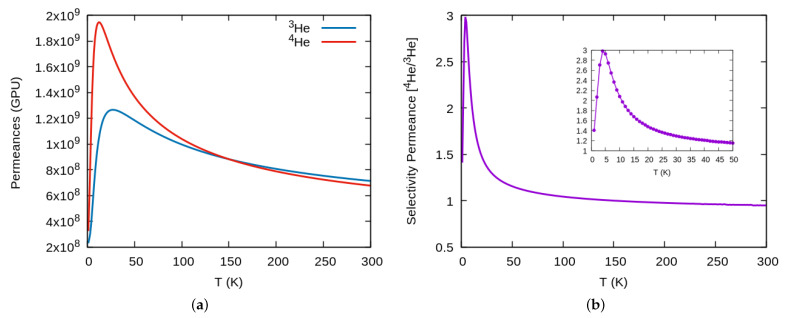
(**a**) Permeances for ${}^{3}He$ and ${}^{4}He$ through graphtriyne at different temperature. (**b**) Permeances selectivity ${}^{4}He{/}^{3}He$.

To finish this section, in Figure 5a we plot the results for the computed permeances in GPU units, which as expected, show a similar behavior to that of rate constants. The corresponding selectivity in Figure 5b, also manifests similar values than that obtained from thermal rates and a maximum yield at about the same temperature. To finish, we would like to remark that it is usually admitted that permeances higher than S(T)>20GPU are industrially appealing, a feature that here is clearly reached for the whole temperature range, up to room temperature.

## 4. Conclusions

We have reported three-dimensional wave packet calculations for the quantum sieving of He atoms through graphtriyne. The transmission probabilities show a strong oscillatory behavior at low energies that leads to isotopic selectivity even though the pore is large enough to become a barrierless process, where very small or no selectivity should be expected. It is suggested that another quantum phenomenon, that of selective adsorption resonances, has a strong influence over the whole temperature range studied. This effect, apart from tunneling or zero point energy effects, has not been considered so far, and we think it is worth to explore. Although it will be difficult to characterize these resonances and to know their features in detail, a procedure similar to the one we described in Reference [57] would help unveil the possibilities of this effect for being used in this kind of materials and processes.

Rate coefficients and permeances are quite large, as corresponds with a material with a large pore size, but exhibiting a maximun and an unexpected selectivity a low temperature. The quantum effects at low energies, just commented, are also responsible of a different behavior to that of graphdiyne regarding the easiness of transmission, since in this case it is the heavier isotope ${}^{4}He$ that it is favored at low temperature instead the lighter ${}^{3}He$, opening new possibilities for the separation of these two isotopes.

Finally, as it has been previously shown [38], the absence of a barrier along the minimum energy path could involve difficulties for approximate treatments based on TST, but in cases like this, more popular treatments making use of classical mechanics could also suffer of large inaccuracies because of the pronounced quantum behavior. It would be then very interesting to see if the next member of the family, graphtetrayne [39,40], recently synthesized and with an even larger nanopore, also shows a similar quantum behavior at low energy and to continue exploring the possibilities of this quantum effect [24,57,58].

## Figures and Tables

**Table 1:** Parameters of atom-atom Improved Lennard-Jones (ILJ) Potential for the interaction of He with graphtriyne layer.

	He-C
$R_{m}$ (Å)	3.663
ϵ (meV)	1.289
β	7.5

## Data Availability

Data is available on the request of corresponding author.

## Appendix A. Details of Wave Packet Calculations

The wave packet is discretized on a grid, with values given in Table A1, and initially is given as a product of a translational gaussian wavepacket [50] for the *z* coordinate, and a plane wave for x,y coordinates along the membrane surface

(A1) $$\Psi \left(r,t=0\right)=G\left(z;z_{0},k_{z0},\alpha \right)\frac{\exp[iK\cdot R]}{{\left(\Delta_{x}\Delta_{y}\right)}^{1/2}}$$

On the one hand, ${\left(\Delta_{x}\Delta_{y}\right)}^{-1/2}\exp\left[iK\cdot R\right]$ is the wave packet depending on the parallel coordinates, where $\Delta_{x}$ = 20.82 Å and $\Delta_{y}$ = 12.02 Å are the lengths of the unit cell. In this work, K=0 to simulate a wave packet approaching to the membrane in the perpendicular direction. The gaussian wave packet is given by

(A2) G(z;z0,kz0,α)=2Im(α)πℏ1/4expiα(z−z0)2/ℏ+ikz0(z−z0)

where $z_{0}$ and $k_{z0}$ are the central values of the wave packet in the position and momentum spaces, respectively, while α is a pure imaginary number that determines the width of the gaussian function.

The grid (and number of points) in the perpendicular coordinate is much larger than in the direction of the graphtriyne layer, to include regions where the He-membrane interaction is negligible, which will be the regions where we will fix the initial wave packet and from where absorbing boundary conditions are applied, Table A2.

**Table A1:** Grid sizes and number of points for the representation of the wave packet. Grid boxes for the parallel coordinates correspond to those of the unit cell of Figure 1a of the main text.

	*x*	*y*	*z*
Box (Å)	(−10.41,10.41)	(−6.010,6.010)	( −30.0, 55.0)
Number of points	128	128	1024

**Table A2:** Parameters of the wave packet propagation, [: - :] indicates the range of values taken in different calculations (definitions specified in the text).

Gaussian Parameters			Time Propagation		Wave Packet Splitting			Flux Surface
$Im\left(\alpha \right)\;\frac{\left[\hbar \right]}{\left[L^{2}\right]}$	$z_{0}$(Å)	$k_{z_{0}}$(Å${}^{-1}$)	δt(fs)	$t_{final}$(ps)	Δt(fs)	$z_{+}$(Å)	β(Å${}^{-2}$)	$z_{f}$(Å)
0.014	40	[3.3–15.0]	0.083	[15.0–18.75]	0.326	[45–55]	[0.005–0.05]	[−2.5–−1.5]

Absorbing boundary conditions [51,52] were applied for the non-periodic coordinate (z) in the regions defined by $z<z_{-}$ and $z>z_{+}$ and with a time period Δt, the wave packet is split into interaction and product wave packets using the damping function $\exp\left[-\beta {\left(z-z_{\pm }\right)}^{2}\right]$. In our particular case, given the grid size, energy and time propagation, there is no absorpion in $z_{-}$, the negative part of the grid along *z*, and the parameters for the damping function are irrelevant. After splitting, propagation is resumed using the interaction portion of the wave packet.

In order to obtain the transmission probability (Equation (3)) it is necessary to compute the stationary wave function and its derivative at the flux surface $z=z_{f}$. This task is performed by accumulating the integrand of the time-energy Fourier transform along the propagation time [53]. Computations run until most of the wave packet gets out of the interaction region, remaining only a small portion in this region (≈1%), for calculations of probabilities shown in the main text. Total propagation times $t_{final}$ range between the values indicated in Table A2, the shortest (largest) times corresponding to the calculations at largest (smallest) energies in the initial wavepacket.

Values of all parameters of the calculations mentioned above are gathered in Table A2. Several convergence checks were carried out by varying these values as well as the number of grid points and the *z* box size.
